# Ektacytometry Analysis of Post-splenectomy Red Blood Cell Properties Identifies Cell Membrane Stability Test as a Novel Biomarker of Membrane Health in Hereditary Spherocytosis

**DOI:** 10.3389/fphys.2021.641384

**Published:** 2021-03-25

**Authors:** M. C. Berrevoets, J. Bos, R. Huisjes, T. H. Merkx, B. A. van Oirschot, W. W. van Solinge, J. W. Verweij, M. Y. A. Lindeboom, E. J. van Beers, M. Bartels, R. van Wijk, M. A. E. Rab

**Affiliations:** ^1^Central Diagnostic Laboratory-Research, University Medical Center Utrecht, Utrecht University, Utrecht, Netherlands; ^2^Department of Pediatric Surgery, University Medical Center Utrecht, Utrecht University, Utrecht, Netherlands; ^3^Van Creveldkliniek, University Medical Center Utrecht, Utrecht University, Utrecht, Netherlands

**Keywords:** hereditary spherocytosis, splenectomy, deformability, ektacytometry, biomarker, red blood cell, hemolytic anemia

## Abstract

Hereditary spherocytosis (HS) is the most common form of hereditary chronic hemolytic anemia. It is caused by mutations in red blood cell (RBC) membrane and cytoskeletal proteins, which compromise membrane integrity, leading to vesiculation. Eventually, this leads to entrapment of poorly deformable spherocytes in the spleen. Splenectomy is a procedure often performed in HS. The clinical benefit results from removing the primary site of destruction, thereby improving RBC survival. But whether changes in RBC properties contribute to the clinical benefit of splenectomy is unknown. In this study we used ektacytometry to investigate the longitudinal effects of splenectomy on RBC properties in five well-characterized HS patients at four different time points and in a case-control cohort of 26 HS patients. Osmotic gradient ektacytometry showed that splenectomy resulted in improved intracellular viscosity (hydration state) whereas total surface area and surface-to-volume ratio remained essentially unchanged. The cell membrane stability test (CMST), which assesses the *in vitro* response to shear stress, showed that after splenectomy, HS RBCs had partly regained the ability to shed membrane, a property of healthy RBCs, which was confirmed in the case-control cohort. In particular the CMST holds promise as a novel biomarker in HS that reflects RBC membrane health and may be used to asses treatment response in HS.

## Introduction

Hereditary spherocytosis (HS) is a heterogeneous group of inherited anemias that originates from defective anchoring of transmembrane proteins to the cytoskeletal network of the red blood cell (RBC). The defective anchoring is predominantly caused by a mutation in the genes coding for ankyrin (ANK1), α-spectrin (SPTA1), β-spectrin (SPTB), band-3 (SLC4A1), or protein 4.2 (EPB42) (Perrotta et al., 2008). These mutations compromise the vertical linkages between the lipid bilayer and the cytoskeletal network, leading to destabilization of the membrane, increased vesiculation and subsequent membrane loss. The progressive membrane loss leads to formation of dense spherical-shaped RBCs (spherocytes) with reduced deformability (Chasis et al., 1988; Eber and Lux, 2004; Huisjes et al., 2018).

The spleen plays an intricate role in the pathophysiology of HS. Normally, this organ functions as a quality control for RBCs. During the 120-day lifespan of healthy RBCs, membrane surface area, surface area-to-volume ratio, and deformability decrease because of release of essentially hemoglobin-free microvesicles. RBCs with increased density and reduced deformability are eventually trapped in the narrow endothelial slits of the spleen, leading to clearance of aged RBCs (Eber and Lux, 2004; Mebius and Kraal, 2005). The compromised vertical linkages in HS accelerate the loss of membrane and deformability, leading to premature destruction of RBCs in the spleen. Therefore, splenectomy is an effective treatment, and removal of the primary site of RBC destruction generally improves clinical symptoms (Musser et al., 1984; Eber and Lux, 2004; Perrotta et al., 2008). Nevertheless, the risks and benefits should be carefully assessed as splenectomy results in a permanently increased risk of infections caused by encapsulated bacteria and long term risk for cardiovascular events (Perrotta et al., 2008; Schilling et al., 2008).

The effects of splenectomy on RBC rheology and RBC related parameters in HS have been studied to a limited extent. It is known that splenectomy improves the RBC count, hemoglobin (Hb) levels, and hematocrit, and that it reduces mean corpuscular hemoglobin concentration (MCHC) and the percentage of reticulocytes (Reliene et al., 2002; Li et al., 2016; Zaninoni et al., 2018; Huisjes et al., 2020). On a cellular level it has been shown that the size of RBCs increases following splenectomy, and that microspherocytes can no longer be detected (Sugihara et al., 1984). However, splenectomy has little effect on correcting the cytoskeletal membrane defect (Reliene et al., 2002). More recent studies have shown that RBC deformability as measured by osmotic gradient ektacytometry was not improved after splenectomy (Zaninoni et al., 2018; Huisjes et al., 2020). An important limitation of these studies was the fact that they compared cohorts of splenectomized and non-splenectomized patients; longitudinal studies on the response to splenectomy of individual HS patients are scarce (Li et al., 2016).

In this study, we investigated individual responses to splenectomy in a group of five HS patients, with particular focus on RBC functional properties as determined by ektacytometry. Our results indicate that the Cell Membrane Stability Test (CMST), which measures the RBCs response to high shear stress, is able to detect substantial functional improvement of the RBC membrane after splenectomy, by showing a partly restored ability to shed membrane, a feature of healthy RBCs. We suggest that the CMST represents a novel biomarker of RBC membrane health in HS, and may be used to assess the efficacy of treatment.

## Materials and Methods

### Patients

Two groups of patients were enrolled in this study. The first group consisted of five patients (one male and four females, aged between 13 and 43 years) diagnosed with HS, and scheduled to undergo splenectomy. Detailed characteristics are provided in Supplementary Table 1. Left-over material of blood collected before splenectomy and at different time points after splenectomy (i.e., 1 week, 1 month and ≥3 months) was used for laboratory measurements. Informed consent was obtained from all patients and/or legal guardians. The second group consisted of a patient cohort of 26 HS patients: 18 non-splenectomized patients, eight patients who underwent splenectomy ≥ 1 year prior to enrollment, and 26 healthy controls (HC). Blood samples of this cohort were obtained after inclusion in the CoMMiTMenT-study which was approved by the Medical Ethical Research Board of the University Medical Center Utrecht, Netherlands (15/426 M) or from anonymized left-over material. Blood from HC individuals was obtained by means of the institutional blood donor service.

### Surgical Procedure

Laparoscopic total splenectomy was performed in all five patients. The patients were positioned in right lateral decubitus. Four trocars were used. The lesser sac was entered and the short gastric vessels were divided. After full mobilization of the spleen, the hilar vessels were controlled by using a linear cutting stapler. The spleen was extracted using a retrieval bag.

### Laboratory Parameters

Routine hematological laboratory parameters were analyzed on an Abbott Cell-Dyn Sapphire hematology analyzer (Abbott Diagnostics Division, Santa Clara, CA, United States).

### Ektacytometry

Deformability of RBCs was measured with the Lorrca (Laser Optical Rotational Red Cell Analyzer, RR Mechatronics, Zwaag, Netherlands). In this ektacytometer, RBCs are exposed to shear stress in a viscous solution (Elon-Iso), forcing the cells to elongate into an elliptical shape. The diffraction pattern that is generated by a laser beam is measured by a camera. The vertical axis (A) and the horizontal axis (B) of the ellipse are used to calculate the elongation index (EI) by the formula (A-B)/(A+B). The EI reflects the deformability of the total population of RBCs.

#### Osmotic Gradient Ektacytometry

Osmotic gradient ektacytometry measurements of RBCs of HC and HS patients before and after splenectomy were obtained using the osmoscan module on the Lorrca according to the manufacturer’s instructions and as described elsewhere (DaCosta et al., 2016; Lazarova et al., 2017). Briefly, whole blood was standardized to a fixed RBC count of 1,000 × 10^6^ and mixed with 5 mL of Elon-Iso (RR Mechatronics). RBCs in the viscous solution (Elon-Iso) were exposed to an osmolarity gradient from approximately 60 mOsmol/L to 600 mOsmol/L, while shear stress was kept constant (30 Pa).

#### Cell Membrane Stability Test

The CMST was performed using the CMST module on the ektacytometer. To perform a CMST, whole blood was standardized to a fixed RBC count of 200 × 10^6^ and mixed with 5 mL of Elon-Iso. In the CMST RBCs are exposed to a shear stress of 100 Pa for 3,600 s (1 h) while the EI is continuously measured. The change in the elongation index (ΔEI) was calculated by determining the median of the first and the last 100 s of the CMST and subsequently calculating the difference between the medians. The ΔEI depicts the capacity of the RBCs to shed membrane and resist shear stress.

Microscopic analysis on a subset of samples was performed with the use of a camera microscope (1/1.8″ Sony CMOS Global IMX265LLR imaging sensor, long working distance VS-Technology 50X Plan LWD, VS-MS-COL tube) which was placed on outside of the rotating cup of the Lorrca. A power-LED flash (415 nm) coupled to a fiber-optic, in bright field illumination, from the inside of the cup into a 45 degrees mirror and diffusor lens directed at the microscope, was used for proper lighting of the RBCs. A flash time of 214 ns was used to get less than 1% motion blur. The rotating cup was modified with 15 thin and small glass windows circumferential in the cup. Images were taken with Image Capture software during a CMST measurement.

### Density Separation

To assess the effect of splenectomy on the composition of the RBC population a density separation was carried out before splenectomy and approximately 1 month after in one HS patient. A total of 20 mL whole blood was placed on top of three layers with different percentage percoll (GE Healthcare) 1.130 ± 0.005 g/mL in eight different columns (2 mL whole blood/column). RBCs were fractioned according to density (i.e., cellular age) using this density gradient of percoll with addition of HEPES, NaCl, KCL, and NaOH as described in detail elsewhere (Rennie et al., 1979). Cells were centrifuged at 1,665 × *g* for 15 min, after which four fractions could be obtained (Supplementary Figure 1A). Fraction 1, containing the RBCs with the lowest density, was present on top of the 59% percoll layer, and was only present and subsequently obtained from the pre-splenectomy blood sample (Supplementary Figure 1A). Because of the limited amount of RBCs in fraction 1 only a subset of measurements could be performed. Fraction 2 was obtained from the top of the 70% percoll layer. Fraction 3 was obtained from the top of the 78% percoll layer. The 4th fraction, containing the most dense RBCs was obtained from the bottom of the tube (Supplementary Figure 1A).

### Digital Microscopy

Peripheral blood smears were analyzed using the CellaVision digital microscope DM96 (software 5.0.1 build11). Analysis was performed using a neuronal network which classifies RBCs based on morphological characteristics such as shape, color, and texture. Spherocytes, microcytes and macrocytes (%) were calculated as a percentage of total RBCs as quantified by the software (Huisjes et al., 2017).

### Statistical Analysis

All the data were analyzed using GraphPad Prism version 8.3.0 for Windows (GraphPad Software, San Diego, CA, United States). A paired *T*-test (two-tailed) was used to assess the values before and after splenectomy. An one-way ANOVA, with post-hoc Tukey analysis was used to assess the differences between HC and HS patients, and non-splenectomized and splenectomized HS patients. In addition, a correlation analysis between RBC related parameters and the change in elongation index (ΔEI) was conducted using the Spearman’s rank correlation coefficient. A *p*-value below 0.05 was considered statistically significant.

## Results

### Routine Hematological Parameters Show a Decrease in RBC Density and Increased RBC Homogeneity After Splenectomy

**FIGURE 1:**
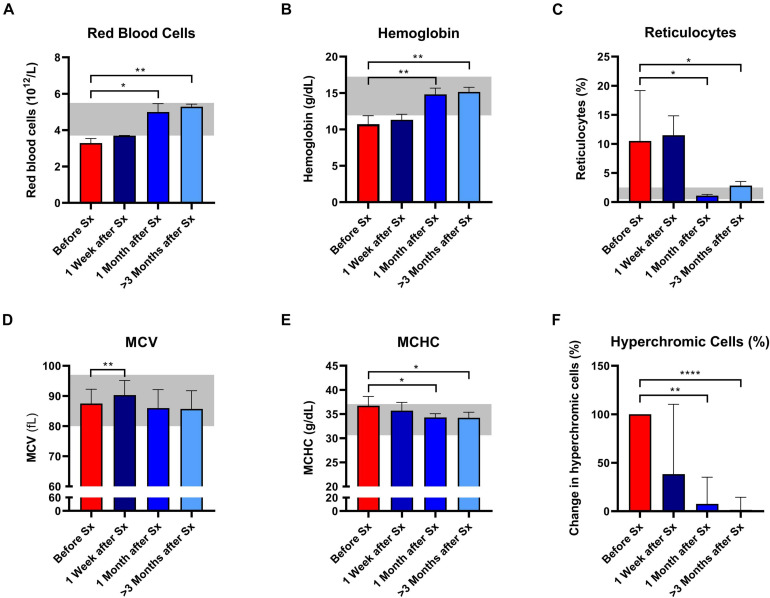
Red blood cell related parameters of patients with hereditary spherocytosis (HS) before and after splenectomy (Sx). Whole blood of HS patients (*n* = 5) was analyzed before and 1 week, 1 and 3 months after splenectomy. **(A)** Red Blood Cells significantly increased after splenectomy. **(B)** Mean hemoglobin values significantly increased after 1 and 3 months after splenectomy. **(C)** Mean values of reticulocytes decreased 1 and 3 months after splenectomy. **(D)** mean values of mean corpuscular volume (MCV) increased significantly 1 week after splenectomy but decreased 1 and 3 months after splenectomy. **(E)** Mean values of mean corpuscular hemoglobin concentration (MCHC) decreased significantly 1 and 3 months after splenectomy. **(F)** Mean change in hyperchromic cells (%) after splenectomy. The laboratory reference ranges (2SD) of the University Medical Center Utrecht (UMCU) are depicted in the light gray area. Error bars represent standard deviation. ****p < 0.0001, ***p < 0.001, **p < 0.01, and *p ≤ 0.05.

Following splenectomy, routine hematology parameters showed a significant improvement in RBC count, Hb, and reticulocyte count after 1 and ≥3 months (Figures 1A–C and Supplementary Table 1). Mean corpuscular volume (MCV) increased in the first week after splenectomy, but returned to pre-surgery levels in the following months (Figure 1D). This suggests that cell volume is not altered by splenectomy. At the same time, we observed a significant decrease in MCHC and the percentage of hyperchromic cells, indicating a decline in RBC density following splenectomy (Figures 1E,F). At the same time, spherocytes and microcytes as assessed by digital microscopy also declined, except for spherocytes in patient 1 (Supplementary Table 1). When RBCs of one HS patient were separated according to density (Supplementary Figure 1A), we noted that the MCHC before splenectomy seemed determined mainly by density fraction 4, containing the most dense RBCs, whereas after splenectomy density fractions 3 and 4 seem to contribute equally to the MCHC (Supplementary Figure 1B), This implies a more homogeneous RBC population after splenectomy, which was also reflected by a more equal distribution of the different fractions after splenectomy (Supplementary Figure 1A). Hyperchromic cells were predominantly present in fraction 4 before splenectomy, and their number decreased substantially after splenectomy (Supplementary Figure 1C).

### Osmotic Gradient Ektacytometry-Derived Parameters Indicate Increased Cellular Hydration After Splenectomy

**FIGURE 2:**
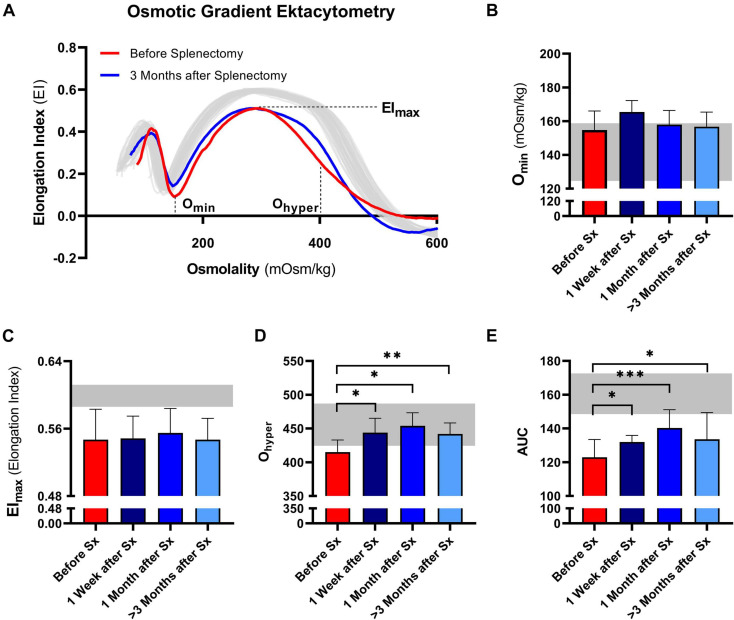
Osmotic gradient ektacytometry curve (Osmoscan) and the corresponding parameters of 5 patients with HS before and after splenectomy (Sx). **(A)** Representative example of the osmotic gradient ektacytometry curve in P03 before and 3 months after splenectomy. The osmotic gradient ektacytometry curves of healthy controls (*n* = 40) are depicted by the gray lines. The changes in **(B)** the surface area to volume ratio of red blood cells (Omin), **(C)** the maximum deformability (EImax), **(D)** the hydration state or cytoplasmic viscosity (Ohyper) and **(E)** the area under the curve (AUC) are depicted above. The HS patients (*n* = 5) were grouped and the results are displayed as the mean (SD) of the combined values. The mean (2SD) of healthy controls (*n* = 74) are depicted in the light gray area **(B–E)**. Error bars represent standard deviation. ***p < 0.001, **p < 0.01, *p ≤ 0.05.

Osmotic gradient ektacytometry was performed to determine the effect of splenectomy on RBC total surface area (EI_max_), surface area-to-volume ratio (O_min_), and RBC hydration state (O_hyper_). In addition, the area under the curve was calculated (Van Vuren et al., 2019). A representative curve of the effect of splenectomy after 1 month is shown in Figure 2A.

Post-splenectomy values for O_min_ and EI_max_ did not change significantly compared to pre-splenectomy values, indicating that after splenectomy HS RBCs still had reduced surface area-to-volume ratio and total surface area (Figures 2B,C, respectively). In contrast, the hydration state or cytoplasmic viscosity (O_hyper_) showed a significant increase toward normal values after already 1 week and this was maintained after 1 and ≥3 months (Figure 2D). This is in line with the decrease in number of hyperchromic cells and MCHC (Figures 1E,F). The increase in O_hyper_ was accompanied by an increase in the AUC, although values remained lower than normal controls (Figure 2E).

Additional osmotic gradient ektacytometry measurements on the different density fractions showed that following splenectomy the variability between the curves from each density fraction is less, again indicating a more homogeneous RBC population (Supplementary Figures 1D,E). These analyses also showed that only O_hyper_ of density fraction 4 improved. Therefore, the increase in O_hyper_ after splenectomy seemed mainly determined by this fraction, containing the most dense RBCs. O_hyper_ of fractions 2 and 3 decreased after splenectomy, most presumably due to a decrease in reticulocytes in both fractions (Supplementary Figures 1D–G).

### The Cell Membrane Stability Test Reveals That HS RBCs Have Regained the Ability to Shed Membrane After Splenectomy, Reflecting Improved Membrane Health

**FIGURE 3:**
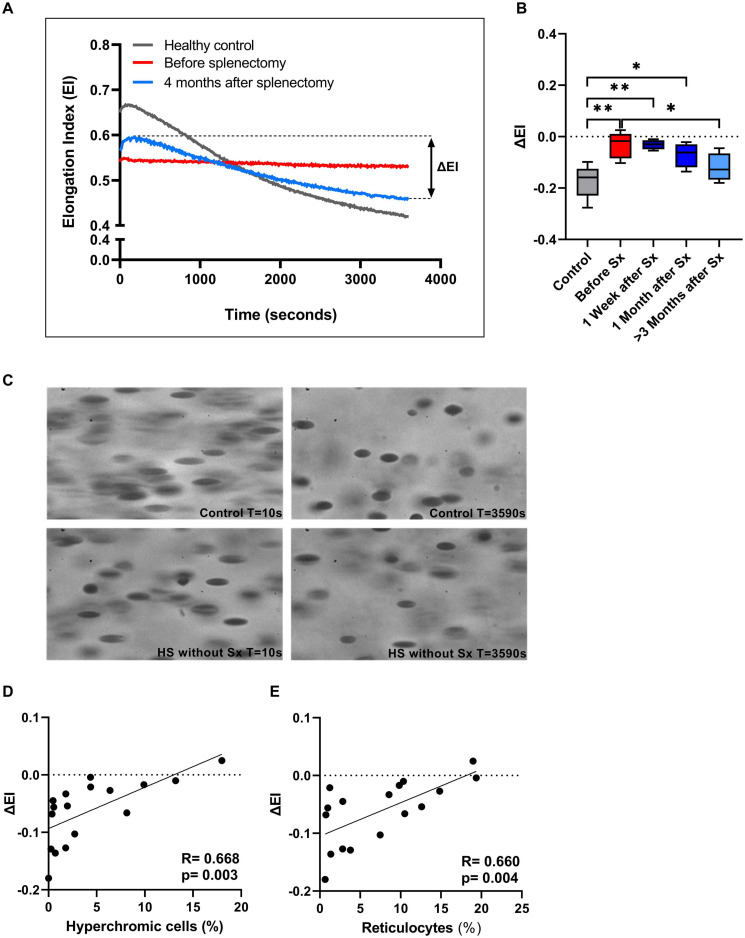
The Cell membrane stability test (CMST) and the calculated parameter (ΔEI) improves after splenectomy in a longitudinal study of 5 HS patients. **(A)** Representative example of the CMST before (red line) and 4 months after splenectomy (light blue line) compared to a healthy control (dark gray line). **(B)** The mean values of ΔEI of HS patients (*n* = 5) before and after splenectomy. **(C)** Microscopic images of the RBCs in the Lorrca during a CMST. Start of the measurement (*T* = 10 s) compared to the end of the measurement (*T* = 3,590 s) in a control and HS patient without splenectomy. **(D)** Correlation between ΔEI and hyperchromic red blood cells. **(E)** Correlation between ΔEI and reticulocytes (%). Error bars represent standard deviation. **p < 0.01, *p ≤ 0.05. Sx, splenectomy; HS, hereditary spherocytosis; T, time; s, seconds.

We next investigated RBC rigidity and its ability to respond to mechanical stress by performing CMST measurements. The CMST exposes RBCs to a high, supraphysiological, shear stress (100 Pa) for the duration of 1 h. Healthy RBCs showed a gradual decrease in deformability under these conditions (Representative curve, Figure 3A), reflected by a negative ΔEI. The loss of deformability likely results from increased vesiculation *in vitro* and consequent membrane loss under shear. In contrast, HS RBCs showed a significantly lower ΔEI before splenectomy compared to HC (*p* < 0.01, Figure 3B; representative curve Figure 3A). This suggests that RBCs of HS patients are more rigid and less able to shed membrane *in vitro* compared to healthy RBCs. After splenectomy, ΔEI increased until it was no longer significantly different from HC ≥ 3 months after splenectomy (Figure 3B). Hence, splenectomy results in a less rigid cell population that has for a large part regained the ability to shed membrane. These findings were strengthened by microscopic analysis of RBCs during the CMST. Figure 3C shows how RBCs obtained from a HC were fully elongated and elliptical at the start of the CMST (*t* = 10 s), then turning into dense and less elongated RBCs at the end of the measurement (*t* = 3,590 s). In contrast, RBCs of an HS patient without splenectomy were already dense and unable to elongate fully at the start of the CMST, remaining like this throughout the measurement. Notably, reticulocyte count and hyperchromic cells both correlated with ΔEI (*r* = 0.660, *p* < 0.01 and *r* = 0.668, *p* < 0.01, respectively, Figures 3D,E), indicating that these cells strongly influence the outcome of CMST measurements.

Additional CMST measurements on the density fractions suggest that the increase in ΔEI post-splenectomy are determined by density fractions 2 and 3 (Supplementary Figure 1H).

### Membrane-Shedding as Measured by the CMST Represents a Novel Pathophysiological Property of HS RBCs

**FIGURE 4:**
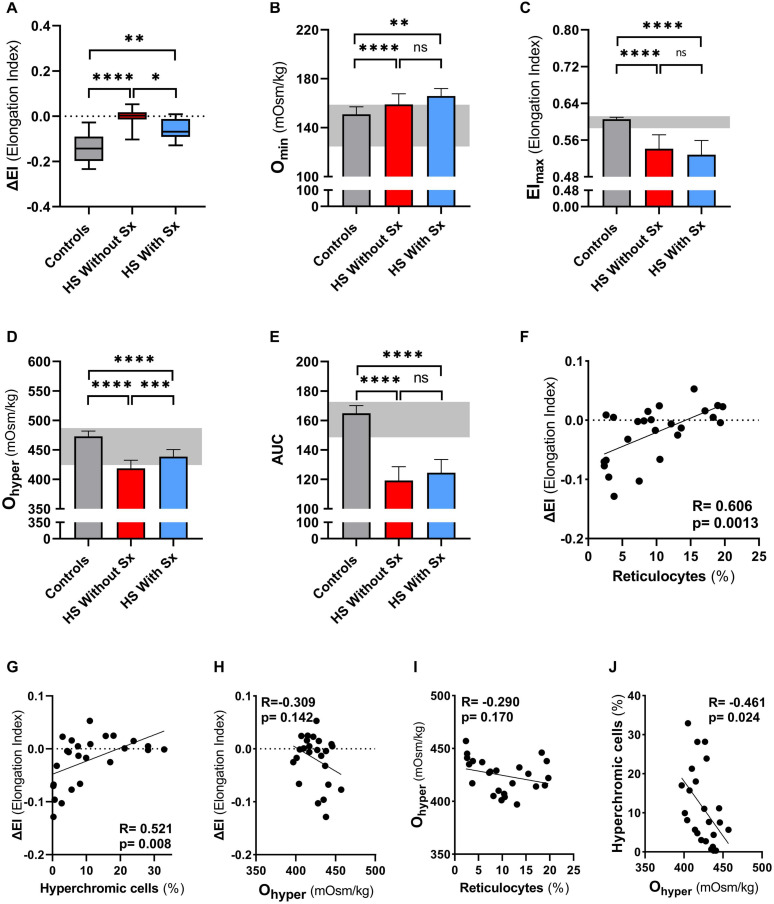
Cell membrane stability test (CMST) and the calculated parameter (ΔEI) shows improvement in splenectomized patients with HS in a case-control study. CMST-derived parameter ΔEI [panels **(A,F,G,H)**], osmotic gradient ektacytometry-derived parameters [panels **(B–E,H,I,J)**], reticulocytes and hyperchromic cells were assessed in 18 non-splenectomized HS patients, 8 non-splenectomized HS patients and 26 healthy controls (HC). **(A)** Mean ΔEI of HS patients without splenectomy compared to patients with splenectomy, both groups were compared to HCs. **(B)** Mean values of Omin are increased in splenectomized patients compared to HCs or non-splenectomized HS. **(C)** Mean values of EImax are decreased in splenectomized HS patient RBCs, compared to HC and non-splenectomized HS RBCs. **(D)** Mean values of Ohyper are significantly different between the 3 groups mentioned above. **(E)** Mean values of AUC show no significant differences between non-splenectomized and splenectomized HS RBCs. **(F)** Linear correlation between reticulocytes (%) and ΔEI of HS patients. **(G)** Linear correlation between hyperchromic cells (%) and ΔEI of HS patients. **(H)** Linear correlation between Ohyper and ΔEI. **(I)** Linear correlation between reticulocytes (%) and Ohyper. **(J)** Linear correlation between hyperchromic cells and Ohyper. Error bars represent standard deviation. ****p < 0.0001, ***p < 0.001, **p < 0.01, *p ≤ 0.05; ns, non-significant. Sx, splenectomy; HS, hereditary spherocytosis; AUC, area under the curve.

To further explore its added value we performed CMST measurements on a large HS cohort consisting of 18 non-splenectomized, 8 splenectomized patients, and 26 HCs. These results confirmed the findings observed in our longitudinal study, showing that the CMST was able to distinguish splenectomized HS patients from non-splenectomized HS patients (*p* = 0.028), in addition to the clear distinction between HS patients in general and HCs (both *p* < 0.001). Similarly, also in this large cohort there was a correlation of ΔEI and reticulocyte count (*R* = 0.606, *p* < 0.01, Figure 4F) and hyperchromic cells (*R* = 0.521, *p* < 0.01 Figure 4G).

We next evaluated osmotic gradient ektacytometry measurements in this case-control cohort. In agreement with our findings in the longitudinal study (Figure 2B), O_min_ was not different in splenectomized patients (Figure 4B). Also EI_max_ and AUC were not significantly different in splenectomized HS patients (Figures 4C,E). In contrast, O_hyper_ was the only parameter that showed improvement when comparing splenectomized to non-splenectomized HS patients (*p* < 0.01, Figure 4D). Furthermore, O_hyper_ correlated with hyperchromic cells although less clear than ΔEI (*R* = −0.461, *p* < 0.05, Supplementary Figure 2), but not with reticulocyte count (Figure 4H). Importantly, ΔEI and O_hyper_ showed no correlation (Figure 4I), suggesting that both biomarkers reflect different features of HS RBCs.

Together, these findings confirm that a decreased ability to shed membrane *in vitro* as measured by the CMST is a novel pathophysiological feature of HS RBCs. It likely reflects membrane health and improves after splenectomy, thereby rendering a novel biomarker that is distinct from the improved density/cell hydration as measured by osmotic gradient ektacytometry.

## Discussion

In the present study, we report on the longitudinal effects of splenectomy in five HS patients. We specifically focused on cellular properties related to membrane health with the use of two different forms of ektacytometry: osmotic gradient ektacytometry and the CMST. In particular the CMST results revealed a novel feature of HS RBCs, i.e., the loss of the ability to shed membrane, and improvement of this *in vitro* cellular property following splenectomy. Membrane-shedding capacity in this test is assessed by the loss of deformability that occurs during prolonged exposure of RBCs to high shear stress. We suggest that improved membrane-shedding capacity after splenectomy reflects improved RBC membrane health, and ΔEI as measured by the CMST may thus serve as a novel clinically relevant biomarker.

The longitudinally observed increase in RBC count, Hb and reticulocyte count after splenectomy corresponds well with results from previous studies where splenectomized and non-splenectomized patient groups were compared (Zaninoni et al., 2018; Huisjes et al., 2020). In addition, our patients also showed a decrease in MCHC directly after splenectomy, which continued to decrease in the following months. This implicates that the internal viscosity or cellular density of HS RBCs is reduced after splenectomy, which is supported by the reduction in the percentage of hyperchromic cells. Little is known about the effect of splenectomy on *in vivo* RBC vesiculation in HS, an important pathophysiological feature, but previous studies demonstrated that RBC vesiculation caused an increase in internal viscosity (MCHC) through shedding of RBC-derived microvesicles (Bosch et al., 1994; Alaarg et al., 2013; Bosman, 2013). Hence, both the decrease in MCHC and hyperchromic cells could indicate that *in vivo* vesiculation of RBCs in HS is reduced after splenectomy. In turn this could explain the observed improvement in ΔEI in the CMST after splenectomy, which reflects improved ability to shed membrane *in vitro*.

More detailed analysis of the longitudinal effects of splenectomy was obtained by osmotic gradient ektacytometry. This technique is generally considered as the gold standard in the diagnosis of HS (DaCosta et al., 2016; Lazarova et al., 2017; Llaudet-Planas et al., 2018; Zaninoni et al., 2018), and its parameters EI_max_, O_min_, and O_hyper_ are considered biomarkers of, respectively, total membrane surface area, surface area to volume ratio, and RBC hydration status. Upon splenectomy most of these parameters were not affected, only O_hyper_ was significantly increased (Figures 2D, 4D). O_hyper_ and MCHC are known to have an inverse correlation with each other (Zaninoni et al., 2018). Both the increase in O_hyper_ and the decrease in MCHC indicate that splenectomy improves the hydration state/intracellular viscosity. In line with this, an increase in the AUC was observed in the longitudinal cohort, whereas AUC remained unchanged after splenectomy in the case-control cohort. Our findings partly contradict with previous studies were both O_hyper_ and AUC remained unaltered after splenectomy (Zaninoni et al., 2018; Huisjes et al., 2020). This could be explained by the non-longitudinal design of these latter studies in which individual differences in response to splenectomy become less apparent.

We next investigated the effect of splenectomy on the ability of HS RBCs to respond to mechanical stress. For this we used the CMST, an ektacytometry based test that was previously used to study membrane stability in HS by studying resealed RBC ghosts (Mohandas et al., 1982; Chasis and Mohandas, 1986). We demonstrate that RBCs from non-splenectomized HS patients show no or only a modest decrease in EI after prolonged exposure to shear stress, in contrast to HC RBCs which display a substantial decrease in EI under these conditions. We hypothesize that HS RBCs from non-splenectomized HS patients are more dense and rigid due to *in vivo* vesiculation that is accelerated by the spleen, and, therefore are less able to shed membrane *in vitro* (Waugh and La Celle, 1980). This is confirmed by the microscopic evaluation of HS RBCs during CMST measurement, which shows that, in contrast to healthy RBCs, HS RBCs do not change morphologically (Figure 3C). Further support for this hypothesis is obtained from the significant correlation of ΔEI and hyperchromic cells (Figure 3D).

Following splenectomy HS RBCs showed an increase in ΔEI (Figure 3B) and after more than 3 months ΔEI was not significantly different compared to HC. This suggests that after splenectomy HS RBCs have regained part of the ability to shed membrane *in vitro*, which may be related to a partly restored ability of HS RBCs for *de novo* synthesis of lipids (Cooper and Jandl, 1969; Sugihara et al., 1984; Takashi and Yoshihito, 1984). The increase in ΔEI could also indicate a change in RBC population due to the absence of the quality control function of the spleen; Instead of shedding micro vesicles *in vivo* in the spleen (i.e., splenic conditioning), this now occurs *in vitro* under the supraphysiological conditions in the CMST. In both cases cellular characteristics as obtained by the CMST measurements indicate an improvement after splenectomy, in a sense that they behave more like normal healthy RBCs. Furthermore, the degree of increase in ΔEI after splenectomy we observed could also be dependent on genetic defect (Ingrosso et al., 1996; Reliene et al., 2002). Our findings were strengthened by CMST results on a large cohort of HS patients that showed that this test was able to discriminate between splenectomized and non-splenectomized HS patients (Figure 4A). This implies that the CMST may represent a novel biomarker of HS, which was further supported by the correlation of ΔEI and % reticulocytes (*r* = 0.66, *p* = 0.004). The ability to distinguish splenectomized patients from non-splenectomized patients can be valuable in an era where different types of splenectomy are explored. Partial splenectomy, either through embolization of splenic arteries or through (laparoscopic) removal of a part of the spleen, might ameliorate symptoms and improve anemia while maintaining splenic phagocytic function (Tchernia et al., 1993; Pratl et al., 2008). However, data of several small studies are inconclusive regarding the remaining immunological capacity of the spleen after partial splenectomy even though hemolysis is decreased (Guizzetti, 2016). Larger studies that also include functional analysis of the spleen and functional analysis of RBCs, i.e., the CMST, are warranted to accurately assess the efficacy of (partial) splenectomy or embolization, and to investigate whether changes in RBC properties or the nature of the underlying molecular defect (Ingrosso et al., 1996; Reliene et al., 2002) contribute to the clinical benefit of splenectomy.

In conclusion, we report on the longitudinal effects of splenectomy on HS RBC characteristics and function as studied by ektacytometry. Our data shows that before splenectomy the HS RBC population is more heterogeneous, cells are more rigid, have increased intracellular viscosity and reduced deformability. Functional analysis of HS RBCs using osmotic gradient ektacytometry and CMST further shows that splenectomy improves the hydration state of HS RBCs and allows cells to regain the ability to shed membrane. In particular the CMST reflects an yet-undescribed distinct RBC characteristic and holds promise as a novel biomarker for membrane health in HS that could be helpful, together with a comprehensive clinical evaluation and appropriate follow-up, to assess the effect of different treatments such as embolization and (partial) splenectomy, and that may be related to clinical severity given the correlation of ΔEI and reticulocyte count. Larger studies are warranted to establish if the CMST can be used to improve the assessment of clinical severity and/or is able to contribute to a better understanding of phenotypic differences in HS.

## Data Availability Statement

The raw data supporting the conclusions of this article will be made available by the authors, without undue reservation.

## Ethics Statement

The studies involving human participants were reviewed and approved by METC University Medical Center Utrecht. Written informed consent to participate in this study was provided by the participants’ legal guardian/next of kin.

## Author Contributions

RH, MB, RW, and MR designed the study. RH, JV, ML, MB, EB, and MR collected clinical and laboratory data. JB, RH, TM, BO, and MR performed laboratory experiments. MCB, RW, and MR analyzed the data and wrote the manuscript. All authors edited the manuscript and approved the final version.

## Conflict of Interest

The authors declare that the research was conducted in the absence of any commercial or financial relationships that could be construed as a potential conflict of interest.
