# Supplementary material for: Ektacytometry Analysis of Post-splenectomy Red Blood Cell Properties Identifies Cell Membrane Stability Test as a Novel Biomarker of Membrane Health in Hereditary Spherocytosis
Source: Front Physiol. 2021 Mar 25;12:641384. doi: 10.3389/fphys.2021.641384 (PMC8027126; doi:10.3389/fphys.2021.641384)
Supplement: Supplementary file 1 [file Data_Sheet_1.PDF]

	Patient 1		Patient 2		Patient 3		Patient 4		Patient 5	
**Patient characteristics**	Pre-Sx	Post-Sx	Pre-Sx	Post-Sx	Pre-Sx	Post-Sx	Pre-Sx	Post-Sx	Pre-Sx	Post-Sx
Age (years)	12		14		13		30		43	
Gene	*ANK1*		*SPTB*		*SPTB*		*SPTA1*		*SLC4A1*	
Mutation	c.5201_5202insTCAG p.(Thr1734fs)		c.4542dup p.(Leu1515fs)		c.647G>A p.(Arg216Gln)		c.3257dup p.(Leu1086fs) c.[5572C>G;6531-12C>T]*		c.2494C>T p.(Arg832Cys)	
Transfusions	0		5		1		0		0	
**Red cell characteristics**										
Hb (g/dL)	8.2	15.5	13.2	15.5	10.7	15.8	9.2	13.1	12.1	15.3
RBC (*10^{12}/L)	3.1	5.8	4.1	5.3	3.3	5.0	2.9	4.2	3.6	4.7
MCV (fL)	76.0	81.0	84.0	82.0	87.5	87.0	92.5	92.5	92.0	93.8
MCHC (g/dL)	35.1	33.3	38.2	35.9	37.2	35.7	35.0	33.5	36.7	35.0
Reticulocytes (%)	19.4	2.3	10.0	2.8	19.0	4.9	7.5	0.6	10.5	1.0
Hypochromic cells (%)	15.7	9.4	N.A.	2.4	3.0	0.8	11.7	36.1	3.1	1.3
Hyperchromic cells (%)	4.4	0.3	9.9	1.8	18.0	0.3	2.7	0.0	8.1	0.5
**Red cell morphology**										
Spherocytes (%)	0.2	8.9	4.8	4.4	1.7	0.1	0.05	N.A.	0.2	0.003
Microcytes (%)	59.8	24.3	51.3	19.3	37.6	0.02	7.9	N.A.	14.2	0.01
Macrocytes (%)	0.2	0.03	0.09	0.0	1.2	0.01	3.5	N.A.	0.3	0.02
**Osmotic gradient ektacytometry**										
Omin	172	166	150	152	160	163	147	147	145	165
EImax	0.547	0.532	0.510	0.510	0.542	0.558	0.582	0.577	0.584	0.591
Ohyper	438	469	401	421	415	438	428	451	404	465

AUC	122.9	139.1	107.6	117.6	117.3	133.2	138.6	154.6	128.3	152.3
**Cell membrane stability test**										
ΔEI	-0.004	-0.07	-0.017	-0.127	0.025	-0.129	-0.103	-0.18	-0.066	-0.056

Legend to Supplementary Table 1.

Detailed overview of patients genotypes, red cell characteristics and morphology, osmotic gradient ektacytometry-derived parameters and Cell Membrane Stability Test results of pre- of post splenectomy (>3 months) blood samples of the patients described in this study.

All patients were heterozygous for the indicated mutations. In addition to the *SPTB* mutation Patient HS02 also carried the Variant of Unknown clinical Significance (VUS) c.3565C>T p.(His1189Tyr) in *SPTA1*.

*Also known as the *SPTA1* alpha-LELY low expression allele.

N.A. Not Available
