# Supplementary material for: Ektacytometry Analysis of Post-splenectomy Red Blood Cell Properties Identifies Cell Membrane Stability Test as a Novel Biomarker of Membrane Health in Hereditary Spherocytosis
Source: Front Physiol. 2021 Mar 25;12:641384. doi: 10.3389/fphys.2021.641384 (PMC8027126; doi:10.3389/fphys.2021.641384)
Supplement: Supplementary file 2 [file Data_Sheet_2.PDF]

Supplemental Figure 1.

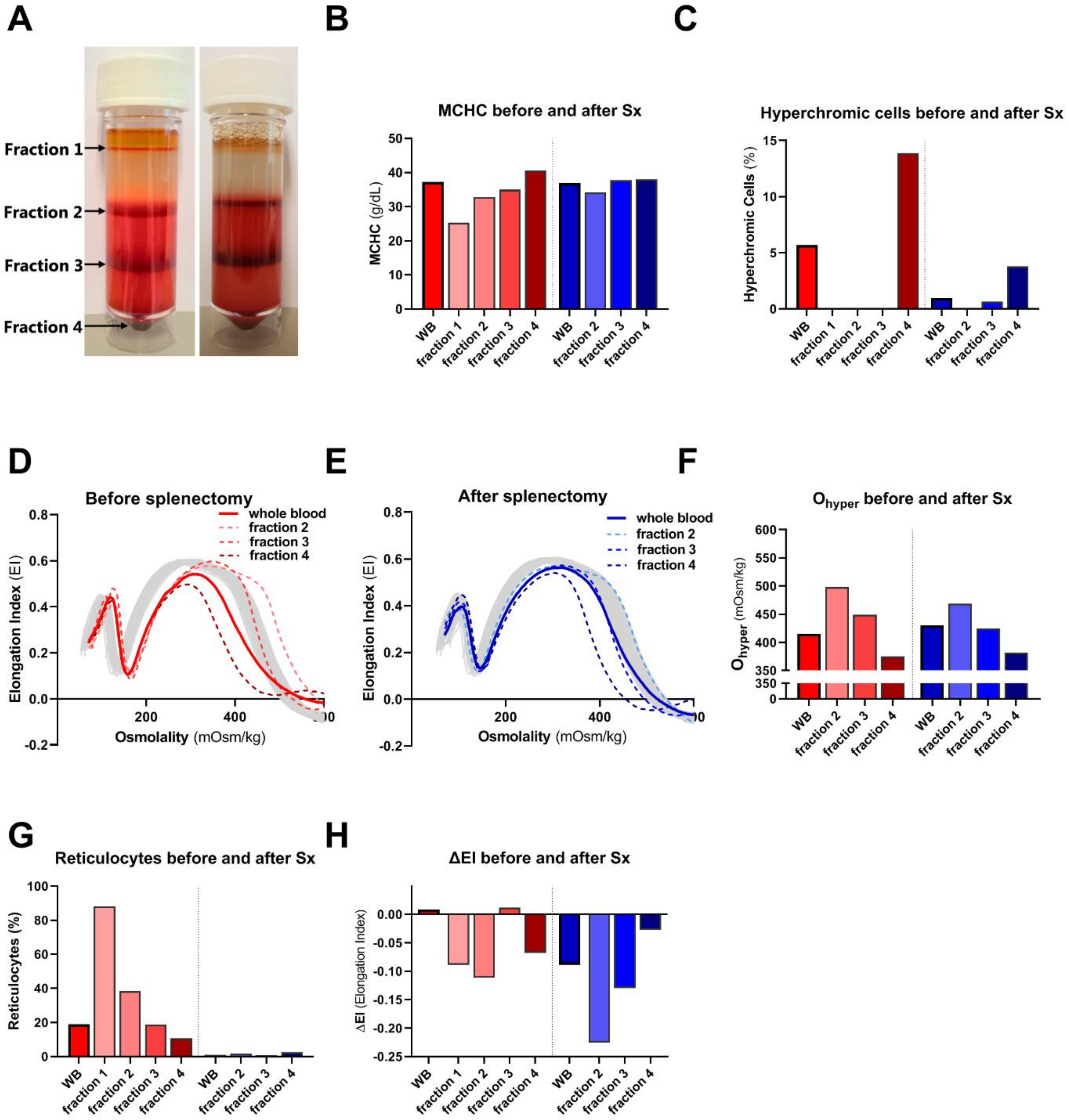

Supplemental Figure 1. Osmotic gradient curves and parameters, CMST parameter (ΔEI) and routine laboratory parameters of fractions with different densities, before and 1 months after splenectomy (Sx). (A) Percoll gradient density separations of a HS patient before (left) and 1 month after splenectomy (right). (B) Mean corpuscular hemoglobin concentration (MCHC) and hyperchromic cells (C) show changes after splenectomy. (D) Osmotic gradient curves of whole blood (solid line) are plotted together with the different density separated fractions (dashed lines) before splenectomy and compared to curves 1 month after splenectomy (E). (F) Ohyper measured in whole blood increases after splenectomy in contrast to fraction 2 that decreases compared to pre-splenectomy values, which is mostly due to decrease in reticulocytes, in all fractions, but most pronounced in fraction 1 and 2 after splenectomy (G). (H) DeltaEI increase substantially after splenectomy which was also found in the different fractions with the exception of fraction 4.

WB, whole blood;
